# Resistance Training for Cyclists: Scientific Evidence and Practical Recommendations

**DOI:** 10.1111/sms.70377

**Published:** 2026-10-03

**Authors:** Raúl de Pablos, Iván R. Sánchez‐Redondo, Lidia B. Alejo, Jesús G. Pallarés, David Barranco‐Gil, Pedro L. Valenzuela

**Affiliations:** ^1^ Department of Sports Sciences. Faculty of Medicine, Health and Sports Universidad Europea de Madrid Madrid Spain; ^2^ Human Performance and Sports Science Laboratory, Faculty of Sport Sciences University of Murcia Murcia Spain; ^3^ Research Institute of the Hospital 12 de Octubre (‘imas12’) Madrid Spain; ^4^ EXERLAB Group, Department of Systems Biology University of Alcalá Madrid Spain

**Keywords:** cycling, endurance, hypertrophy, performance, strength training

## Abstract

Growing evidence supports strength or resistance training (RT) as an effective method for enhancing cycling performance, but many cyclists remain reluctant to incorporate it due to perceived detrimental effects, such as excessive residual fatigue and unwanted hypertrophy. Furthermore, controversy persists regarding optimal training methodologies. This narrative review aims to summarize current evidence on the effects of RT on cycling performance, as well as to address its potential impact on body composition and bone health. We also provide evidence‐based recommendations on best practices for RT implementation. Overall, the addition of 1–2 weekly sessions of RT with moderate‐to‐high loads (60%–85% 1RM), 2–4 sets per exercise across 3–5 compound lower‐body movements, and limited intra‐set fatigue (10%–20% velocity loss) has proven beneficial for improving key cycling performance indicators. Recent evidence also suggests that high‐load on‐bike torque training may serve as an effective strategy when conventional RT is not feasible. When implemented as described, RT does not appear to negatively affect performance through unwanted hypertrophy. Although further research is warranted, RT also emerges as a potentially effective strategy to counteract the low bone mineral density commonly observed in competitive cyclists. However, the magnitude of these benefits largely depends on precise programming, particularly intensity, proximity to failure, and temporal separation from endurance training sessions. In summary, current evidence supports the role of RT for improving both health and performance in cyclists, but further research is warranted to determine the optimal training parameters to maximize its benefits.

## 1 Introduction

Cycling is a complex sport in which performance is determined by both extrinsic factors (e.g., race profile, weather conditions, altitude) and individual variables (e.g., mental, physical, physiological, and technical attributes) [1, 2, 3, 4]. Among the latter, performance is essentially determined by the ability to sustain high power output values (i.e., the product of the torque applied to the crank and pedaling cadence), which is in turn conditioned by other physiological factors such as maximal aerobic capacity, fractional utilization, or economy/efficiency [1, 2, 3, 4]. While enhancing endurance capacity has traditionally been the primary focus of cycling training, recent evidence underscores that the ability to generate and sustain high levels of torque is a critical determinant of competitive success [5, 6]. For instance, our research group observed that differences in power values between cyclists of different performance levels (e.g., UCI WorldTeam, Pro‐Team, or Under‐23) were mostly due to differences in torque rather than cadence [5]. Similarly, the ability to sustain high torque values under fatigue, a key component of durability, has been identified as a differentiating factor of top‐level cyclists [6].

Given the central role of torque for power output production, attaining high levels of lower‐body muscle strength is likely of relevance for cycling performance. Growing evidence indeed supports the association of muscle strength indicators and cycling performance. In elite cyclists, knee extension maximum voluntary torque and rate of torque development are positively associated with W′—a key marker of anaerobic capacity—and critical power [7, 8] while quadriceps muscle volume has been identified as a determinant of peak power output (PPO) [9, 10]. More recently, Javaloyes et al. [11] analyzed a large cohort of cyclists from the same national team and observed that maximum strength (1‐repetition maximum) on the back squat exercise was positively associated with maximal aerobic power expressed in absolute units and relative to body mass.

These associations suggest that enhancing lower‐body muscle strength may be a meaningful lever for improving cycling performance, supporting the rationale for incorporating resistance training (RT) into cyclists' programs. However, despite growing evidence in favor of this strategy [12], many cyclists remain reluctant to do so [13]. Indeed, a recent study involving 147 professional cyclists reported that while the vast majority engage in two RT sessions per week during the off‐ and pre‐season (~90% overall, with higher adherence in women than men), fewer than half (48%) maintain any RT during the competitive season [13]. Persistent concerns over excessive muscle mass gains—reported by 45% of respondents—and practical barriers such as travel, fatigue, and racing schedules were identified as the primary obstacles to consistent practice.

Previous reviews have tried to summarize the effects of RT on cycling performance. For instance, two narrative reviews, published more than 10 years ago, synthesized existing evidence on the effects of RT on cycling performance, as well as on the potential mechanisms involved. However, a large number of studies have been published since then. More recently, a meta‐analysis quantitatively summarized the benefits of RT on specific cycling performance indicators (e.g., time trial performance, maximum oxygen uptake [VO_2_max], ventilatory thresholds) [12]. Nonetheless, numerous research gaps remain, such as the effects of strength training on specific cyclists' populations (e.g., female cyclists, master cyclists) or on other relevant indicators (e.g., body composition). Moreover, the influence of RT parameters (e.g., frequency, intensity, proximity to failure) on cycling performance also remains to be determined. It is also worth noting that, in recent years, the so‐called “torque” training has gained popularity as a potential alternative to traditional gym‐based RT [14, 15, 16, 17]. However, to the best of our knowledge no previous review has attempted to summarize evidence on this training type.

Under this context, this narrative review aims to summarize current evidence on the effects of RT in cyclists, with a main focus on cycling performance but also addressing its impact on body composition and bone health. Furthermore, we aim to provide evidence‐based recommendations on best practices for RT, including an appraisal of on‐bike RT as an emerging alternative to conventional gym‐based exercise. For this purpose, we performed a non‐systematic search in PubMed (from inception to May 2026) using the terms “cycl*” and “triathl*” combined with others related to the intervention of interest (i.e., “resistance training,” “strength training,” “concurrent training,” “torque training”). This search was complemented by manual screening of the reference list of retrieved articles and recent reviews on the topic. Given the non‐systematic nature of the selection strategy, this narrative review is prone to selection bias. To ensure a high level of evidence, we prioritized randomized and non‐randomized controlled trials conducted in cyclists assessing the effects of gym‐based or on‐bike torque training on cycling performance, body composition, or bone‐health outcomes. In order to provide practical recommendations on how to perform RT, evidence could be derived from studies conducted in non‐cyclist populations when studies in cyclists were not available. Studies derived from conference proceedings and non‐peer‐reviewed publications were excluded.

## 2 Effects of Resistance Training on Cycling Performance

Growing evidence consistently supports that incorporating traditional gym‐based RT (mainly with moderate‐high loads and multi‐joint lower‐body exercises; see section on *Practical Applications* for more details) provides significant benefits in cycling performance. A summary of some of the main trials assessing the effects of RT in cycling performance is shown in Table 1.

**TABLE 1:** Characteristics of main trials assessing the effects of conventional “gym‐based” resistance training in cyclists.

Authors	Total sample (groups)	Resistance training intervention			Main results
		Duration (frequency)	Exercises	Load	
Bishop et al. (1999) [18]	21 [21F] (14 RT; 7 C)	12 weeks (2 sessions/week)	Squat	3–5 sets of 2–8 reps; 80%–95% 1RM progressive	↑1RM*
Bastiaans et al. (2001) [19]	14 [0F] (6 RT; 8 C)	9 weeks (2 sessions/week)	Squat, leg press, single‐leg step up, leg pull, core	4 sets of 30 reps; ~50% 1RM	↑Wmax in the first 4 weeks* ↑ Mean PO in a 60‐min TT in the first 4 weeks* **↑Wmax in the first 9 weeks+** ↑ Mean PO in a 60‐min TT in the first 9 weeks* ↓30‐s Mean PO in C*
Paton et al. (2005) [20]	18 [0F] (9 RT + Sprint; 9 C)	6 weeks (2 sessions/week)	Single‐leg explosive jump, high‐resistance cycling sprints	3 sets of 20 reps with bodyweight and 5 reps of 30″ sprint training	↑ Mean PO in a 1‐km TT* ↑ Mean PO in a 4‐km TT* ↑PPO* ↑ Mean PO at LT* ↓Oxygen cost*
Jackson et al. (2007) [21]	14 [4F] (9 RT; 5 C)	10 weeks (3 sessions/week)	Squat, machine leg curls, machine leg press, machine single‐leg step‐ups	First week familiarization with 3 sets of 10 reps; ~50% 1RM. Remainder of the period with 4 sets of 4 reps; ~85% 1RM	↑1RM*
Levin et al. (2009) [22]	14 [0F] (7 RT; 7 C)	6 weeks (3 sessions/week)	Upper and lower‐body exercises focused on hypertrophy (i.e., unilateral leg press, abdominal crunch), strength (i.e., squat) and explosive exercises (i.e., clean grip deadlift)	3–4 sets of 5–12 reps; 75%–90% 1RM	↑ 1RM* ↓ Time in a 1‐km TT*
Hausswirth et al. (2010) [23]	14 [0F] (7 RT; 7 C)	5 weeks (3 sessions/week)	Leg extension, leg press, hamstring curl, leg curl	3–5 sets of 3–5 reps; 87%–97% 1RM	↑ 1RM after 2 h pedaling ↑ MIF* ↑ Stabilized EMGi (VL) after 2 h pedaling*
Rønnestad et al. (2010a) [24]	20 [2F] (11 RT; 9 C)	12 weeks (2 sessions/week)	Half squat, unilateral leg press, single‐leg hip flexion, ankle plantar flexion	3 sets of 4–10 reps; 75%–90% 1RM progressive	↑ CSA* ↑ MIF* ↑ PPO in the Wingate test* ↓ Fatigue index in the Wingate test* ↑ VO_2_max ↑ Wmax* ↑ Mean PO in a 40‐min TT* **↑ Mean PO at 2 mmol L** ^−1^*
Rønnestad et al. (2010b) [25]	12 [1F] (6 RT; 6 C)	25 weeks (2 sessions/week)	Half squat, unilateral leg press, single‐leg hip flexion, ankle plantar flexion	1–3 sets of 4–10 reps; 75%–90% 1RM progressive	↑ 1RM* ↑ CSA* ↑ Wmax* ↑ Mean PO in the Wingate test ↑ PPO in the Wingate test* ↑ VO_2_ ↑ Mean PO in a 40‐min TT* ↑ Mean PO at 2 mmol L^−1^
Sunde et al. (2010) [26]	13 [3F] (8 RT; 5 C)	8 weeks (3 sessions/week)	Half squat in a Smith‐machine	4 sets of 4 reps; 90% 1RM	↑ 1RM* ↑ RFD* ↑ Cycling economy* ↑ Work efficiency* **↑ Mean PO at 70% VO**_**2**_ max* **↑ TTE at VO**_**2**_ max*
Aagaard et al. (2011) [27]	14 [0F] (7 RT; 7 C)	16 weeks (3 sessions/week)	Isolated knee extension, incline leg press, hamstring curls, calf raise	4 sets of 5–12 reps; 67%–87% 1RM progressive	↑ MVC* ↑ RFD* ↑ 5‐min TT performance ↑ 45‐min TT performance* ↑ IIA fibers* ↓ IIX fibers* ↑ Lean body mass*
Rønnestad et al. (2011) [28]	20 [2F] (11 RT; 9 C)	12 weeks (2 sessions/week)	Half‐squat in a Smith‐machine, unilateral leg press, single‐leg hip flexion, toe raise	3 sets of 4–10 reps; 75%–90% 1RM progressive	↑ 1RM* **↓ VO**_**2**_, **HR**, lactate and RPE in prolonged cycling* ↑ Mean PO in a 5‐min TT after 185 min pedaling*
Hansen et al. (2012) [29]	18 [2F] (10 RT; 8 C)	12 weeks (2 sessions/week)	Half squat, unilateral leg press, single‐leg hip flexion, ankle plantar flexion	3 sets of 4–10 reps; 75%–90% 1RM progressive	↑ 1RM* ↑ Mean PO in a 5‐min TT* ↑ Peak positive crank torque* ↓ Phase with negative crank torque*
Psilander et al. (2015) [30]	19 [0F] (10 RT; 9 C)	8 weeks (2 sessions/week)	Leg press	5 sets of 10–15 reps; 65%–75% 1RM	↑ 1RM* ↑ PPO in the Wingate test ↑ TTE at VO_2_max ↑ VO_2_max
Rønnestad et al. (2015) [31]	16 [0F] (9 RT; 7 C)	25 weeks (1–2 sessions/week)	Half squat, unilateral leg press, standing single‐leg hip flexion, ankle plantar flexion	3 sets of 4–10 reps; 75%–90% 1RM progressive	↑ MIF* ↑ Body mass ↑ Lean lower‐body mass* ↑ PPO in the Wingate test. ↑ Wmax* ↑ Mean PO at 4 mmol L^−1^. ↑ Mean PO in a 40‐min TT* ↑ Peak torque during pedal stroke*
Vikmoen et al. (2016) [32]	19 [19F] (11 RT; 8 C)	11 weeks (2 sessions/week)	Half squat in a Smith‐machine, unilateral leg press, single‐leg hip flexion, ankle plantar flexion	3 sets of 4–10 reps; 75%–90% 1RM progressive	↑ 1RM* ↑ CSA* ↑ PPO in the Wingate test* ↑ Mean PO in the Wingate test* ↑ Mean PO in a 40‐min TT ↓ VO_2_ and ↑ cycling economy in a 40‐min TT ↑ IIA fibers* ↓IIX fibers*
Rønnestad et al. (2017) [33]	20 [4F] (12 RT; 8 C)	10 weeks (2 sessions/week)	Half squat, unilateral leg press and hip flexion, ankle plantar flexion	3 sets of 4–10 reps; 75%–90% 1RM progressive	↑ MIF* ↑ Squat jump* ↑ PO in the Wingate test*
Beattie et al. (2017) [34]	15 [0F] (6 RT + URT + PL; 9 C)	20 weeks (2 sessions/week)	Upper and lower‐body exercises focused on strength (i.e., Trap‐bar deadlift, split squat), plyometric exercises (i.e., squat jump)	2–3 sets of 3–12 reps; Bodyweight, 75%–90% 1RM	↑ 1RM* ↑ 6‐s Mean PO* ↑ Wmax* ↑ Body mass* ↑ Leg lean mass*
Vikmoen et al. (2017) [35]	19 [19F] (11 RT; 8 C)	11 weeks (2 sessions/week)	Half squat, unilateral leg press, hip flexion, and ankle plantar flexion	3 sets of 4–10 reps; 75%–90% 1RM progressive	↑ 1RM* ↑ Leg lean mass* ↑ IIA fibers* ↑ CPT2 and LDHB gene* **↓VO**_**2**_ in prolonged cycling* ↑ Mean PO in a 5‐min TT ↑ Distance in 5‐min run*
Del Vecchio et al. (2019) [36]	25 [0F] (9 RT + ST; 7 ST; 9 C)	12 weeks (2 sessions/week)	Upper and lower‐body exercises focused on hypertrophy (i.e., leg extension, chest press), strength (i.e., seated hip flexion), plyometric (i.e., box jump) and explosive exercises (i.e., single‐leg press throw); cycling sprints (standing and flying starts)	1–4 sets of 2–10 reps; Bodyweight, 20%–90% 1RM; maximal sprints at 90–110 rpm	↑ Mean PO in the Wingate test in both experimental groups* ↑ Lower limb lean mass in both experimental groups ↑ 200‐m performance in RT + ST*
Gil‐Cabrera et al. (2021) [37]	20 [0F] (11 RT; 9 VBRT)	8 weeks (2 sessions/week)	Squat, hip thrust, lunge	RT: 3 sets of 4–10 reps; 75%–90% 1RM progressive. VBRT: 3 sets of maximum repetition to 10% VLoss; Optimal load (~65% 1RM)	↑ 1RM in both ↑ Muscle mass in both ↑ Mean PO at RCP in both ↑ Performance in an 8‐min TT in both
Luckin‐Baldwin et al. (2021) [38]	25 [6F] (14 RT; 11 C)	24 weeks (2 sessions/week)	Half squat, glute hamstring raises, hip thrust, unilateral leg press and seated calf raises, hip flexion, hip abduction	3–5 sets of 1–12 reps; 75%–85% 1RM progressive	↑ 1RM ↑ Cycling economy ↑ Running economy
Montalvo‐Pérez et al. (2021) [39]	17 [17F] (8 RT; 9 VBRT)	6 weeks (2 sessions/week)	Squat, hip thrust, split squat	RT: 3 sets of 4–8 reps; 80%–90% 1RM progressive. VBRT: 3 sets of maximum repetition to 10% VLoss; Optimal load (~65% 1RM)	↑ 1RM both in both* ↑ Muscle power in both ↑ Mean PO in an 8‐min TT
Ji et al. (2022) [40]	20 [3F] (7 RT; 7URT; 6C)	10 weeks (2 sessions/week)	RT: Leg press, leg extension, and leg curl. URT: Leg press, leg extension, and leg curl	4 sets of 4–10 reps; 75%–90% 1RM progressive	↑ Body mass only RT* ↑ Lean body mass only RT* ↑ MIF in both experimental groups ↑ TTE at VT2 in both experimental groups ↑ Cycling economy only RT*
Jones et al. (2022) [41]	13 [0F] (6 RT + E; 7 RT + HIIT)	8 weeks (2 sessions/week)	Squat, single‐leg split squat, calf raise	3 sets of 4–10 reps; 75%–90% 1RM progressive	↑ 1RM in both experimental groups ↑ CMJ in both experimental groups **↑ VO**_**2peak**_ only RT + E*
Sitko et al. (2024) [42]	24 [0F] (12 RT; 12 C)	12 weeks (2 sessions/week)	Half squat, unilateral leg press, single‐leg hip flexion and ankle plantar flexion	3 sets of 6 reps; 85% 1RM	↑ 5‐s Mean PO* ↑ 1‐min Mean PO* ↑ Mean PO in a 5‐min TT* ↑ Mean PO in a 20‐min TT*
Barranco‐Gil et al. (2025)^a^ [16]	25 [0F] (12 RT; 13 C)	10 weeks (2 sessions/week)	Squat	5 sets of 7 reps; 70% 1RM	↑ 1RM* ↑ Pedaling‐specific strength* ↑ Mean PO and PPO in the Wingate test* ↑ CSA*
Pallares et al. (2025)^a^ [17]	25 [0F] (12 RT; 13 C)	10 weeks (2 sessions/week)	Squat	5 sets of 7 reps; 70% 1RM	↑ MAP* ↑ CSA* ↑ 1RM*

*Note:* Results in the Main results column are those that showed significant within‐group changes, and those in bold font and marked with an asterisk also showed significant between‐group differences (*p* < 0.05).

Abbreviations: 1RM, one repetition maximum; C, control; CMJ, counter movement jump; CSA, cross‐sectional area; E, endurance; EMGi, integrated electromyography; F, female; FTP, functional threshold power; G, group; HIIT, high intensity interval training; HR, heart rate; LT, lactate threshold; MAP, maximal aerobic power; MIF, maximal isometric force; min, minutes; MVC, maximal isometric quadriceps muscle strength; *n*, sample size; NR, not reported; PL, plyometric training; PO, power output; PPO, peak power output; RCP, respiratory compensation point; RFD, rate of force development; RT, resistance training; ST, sprint training; TS, total sessions; TT, time trial; TTE, time to exhaustion; URT, unilateral resistance training; VBRT, velocity‐based resistance training; VL, vastus lateralis; VLoss, velocity loss; VT2, second ventilatory threshold; Wmax, peak power.

^a^ These studies pertain to the same trial.

Classic studies in this field, with training programs based on multi‐joint lower‐body exercises performed with high loads, have shown robust increases in maximum strength and power production parameters, accompanied by improvements in pedaling economy and performance in prolonged time‐trial efforts and Wingate‐type sprint tests, without substantial changes in maximal oxygen uptake (VO_2_max) [24, 26, 27]. For instance, Rønnestad et al. [24] observed that the addition of 2 weekly sessions of heavy RT based on half squats performed at 85%–90% of 1RM (4RM) during a 12‐week preparatory period improved not only maximal strength but also 40‐min time‐trial performance and peak and mean sprint power compared with endurance training alone. Importantly, these improvements with RT occurred despite no significant differences between groups in VO_2_max gains, which might suggest that the performance gains induced by RT could be mainly mediated by neuromuscular adaptations. Similarly, in a study involving young high‐level cyclists, Aagaard et al. [27] observed that adding RT to regular endurance training was associated not only with increases in maximal strength and rate of force development (RFD) but also with improvements in 20–40 min time trial performance and transitions in muscle fiber phenotypes from type IIX to IIA, again without significant changes in VO_2_max. Consistent with these findings, Sunde et al. [26] further demonstrated that heavy RT improved gross efficiency during submaximal cycling, providing additional mechanistic support for the economy‐related benefits. Two recent studies derived from one randomized controlled trial have further corroborated these findings, showing that full squat‐based programs (2 sessions/week, 70% 1RM) improve sprint power and maximal aerobic power in well‐trained cyclists [16, 17].

Evidence in support of these beneficial effects has accumulated in the last decade. A recent meta‐analysis by Llanos‐Lagos et al. [12] synthesized 17 controlled trials in which heavy RT (1–3 sessions per week) was integrated with regular cycling training for periods of 5–25 weeks, and its results indicate small‐to‐moderate improvements in specific performance indicators (i.e., time trials and tests to exhaustion), as well as in efficiency/economy and anaerobic power. In turn, changes in VO_2_max, maximal aerobic power (MAP), and other indicators such as maximal lactate steady state (MLSS) and ventilatory thresholds did not reach statistical significance [12]. Evidence also shows that the benefits of adding RT extend to conditions in which performance is limited by accumulated fatigue (i.e., the so‐called durability). It has been observed that after ~3 h of submaximal cycling at moderate intensity, cyclists who added heavy RT to their usual endurance programs exhibited lower ratings of perceived exertion, reduced blood lactate concentration, and a more stable heart rate response during the final stages, and subsequently produced greater power outputs in 5‐min maximal efforts and 30‐s sprint tests, suggesting improved durability [28, 29].

Although less extensive, evidence in female cyclists converges with the findings observed in men. In well‐trained competitive female cyclists, adding 2 weekly heavy RT sessions for 11 weeks increased not only maximal strength (1RM), but also 40‐min time‐trial performance while reducing the fractional utilization of VO_2_max and improving pedaling economy, again without significant changes in VO_2_max [32]. In addition, in well‐trained female endurance athletes, heavy RT improved performance in a 5‐min all‐out test performed after ~3 h of submaximal exercise, indicating enhanced ability to sustain high power output following prolonged prior work (i.e., improved durability) [35].

## 3 Effects of Resistance Training on Body Mass and Body Composition

Traditionally in endurance sports, and particularly in cycling, concerns have been raised regarding the potential increase in body mass associated with RT and its possible negative impact on performance, especially during uphill climbing where the influence of gravity increases. This perception remains prevalent among professional cyclists. As mentioned above, in a recent survey of 147 UCI professional road cyclists, 45% reported being concerned about gaining excessive muscle mass as a consequence of RT [13]. However, available evidence suggests that traditional RT does not typically cause meaningful increases in total body mass in cyclists, while it can improve absolute power and preserve or even increase relative power output [43].

Classical studies reinforce this idea. In competitive cyclists, a maximum strength block based on half squats (4 × 4 at 4RM) for 8 weeks produced significant improvements in pedaling economy without changes in body mass [26]. Although power was reported in absolute terms, the absence of changes in body mass indicates that improvements in performance were not accompanied by a weight penalty. Similarly, in another study in well‐trained cyclists, 12 weeks of high‐load RT increased thigh cross‐sectional area and peak power in Wingate; of note, the gain in relative power was greater than that of the control group, indicating that any change in body mass was insufficient to limit relative performance [24]. Another study in elite cyclists showed small seasonal increases in lean lower‐body mass among cyclists who combined RT and endurance training during 25 weeks compared with those who undertook only endurance training, but the former improved sprint relative power and relative power output in a 40‐min time trial, implying performance improvements despite minimal changes in total body mass [31, 33].

Studies that analyze not only body mass but also body composition provide a key insight: body recomposition may be more relevant than absolute weight. In professional cyclists, both a traditional heavy RT program and one based on the so‐called “optimal load” (i.e., training with the load that maximizes external power output) increased muscle mass and reduced fat mass without changing total body mass, while improving strength/power and 8‐min time trial performance [37]. In master cyclists, a combined strength and sprint program increased lean mass in the lower‐body and improved sprint variables, suggesting that regional muscle gain can translate into greater power output with minimal impact on total weight [36]. This pattern has been confirmed in more recent randomized controlled trials, in which squat‐based RT increased quadriceps cross‐sectional area without inducing meaningful changes in total body mass [16, 17]. Indeed, quadriceps muscle volume has been identified as an important predictor of torque production capacity and even PPO [9, 10]. Similarly, a study in female cyclists found that the improvement observed in a 40‐min time trial after 11 weeks of RT was positively correlated with the increase in quadriceps cross‐sectional area [32].

Taken together, the available evidence suggests that the addition of RT in cyclists elicits minimal increases in body mass, and although it can increase lower‐body muscle mass, these increases are associated with even larger increases in power production capacity, thereby yielding improvements not only in absolute power, but also in relative power production.

## 4 Beyond the Muscle: Effects of Resistance Training on Bone Health

Apart from its beneficial effects on performance, RT has been proposed as a beneficial strategy for cyclists' health, and particularly for mitigating the side effects of prolonged cycling on bone mineral density (BMD). Growing evidence suggests that, as compared to other sports, cycling does not confer any significant osteogenic benefit due to the long hours spent in a weight‐supported position on the bike [44]. Indeed, low BMD is highly prevalent in elite cyclists (affecting 45% and 64% of female and male cyclists, respectively) [45], and further reductions in BMD are observed within just one season [46].

Although different factors can contribute to this low BMD (e.g., low‐impact exercise, low energy availability, nutrient inadequacy), integrating bouts of heavy RT or high‐impact loading might counteract these adverse effects [47]. A cross‐sectional observational study in 40 cyclists revealed that those who performed RT showed a higher BMD at different body sites including lumbar spine, hip, and femoral neck [48]. Some trials have also found beneficial effects of RT on BMD. Valenzuela et al. [49] showed that 7 weeks of heavy RT (2 sessions per week including back squat, lunges and hip thrust) induced increases in total bone mineral content in professional male cyclists. A more recent study in elite male and female cyclists showed that a combination of plyometric training (i.e., jumps) and collagen supplementation preserved BMD in the femoral neck, whereas a reduction was observed in a control group that maintained their habitual training routine [50]. Despite its potential beneficial effects, evidence remains preliminary, and indeed other observational studies have found a low BMD in cyclists despite performing RT (although no details were given on the RT program performed) [51]. Further research is therefore warranted to confirm whether RT can mitigate the adverse effects of cycling on bone health.

## 5 Practical Recommendations for Implementing Resistance Training in Cyclists

While current evidence strongly supports the integration of RT for cyclists, skepticism remains prevalent, particularly regarding improper implementation. To date, most studies assessing the effects of RT on cycling performance have implemented quite homogenous training regimes. For instance, as summarized in the meta‐analysis by Llanos‐Lagos et al. [12], most studies add 1–3 strength sessions per week to the cyclists' habitual endurance training, with a predominance of multi‐joint lower‐body exercises (squats or half squats, leg presses, lunges or split squats, hip thrusts, knee extensions and flexions, plantar flexion) with loads ≥ 75%–90% 1RM and long breaks between sets (2–3 min). Notwithstanding, concerns often center on the interference effect, where excessive fatigue or delayed onset muscle soreness from strength sessions may compromise subsequent sport‐specific endurance bouts. Furthermore, the power‐to‐weight ratio is a critical performance determinant in cycling, and consequently, excessive muscle hypertrophy may be a detrimental adaptation. Optimal programming of RT can mitigate these adverse effects.

### 5.1 Exercise Selection

As mentioned above, most studies to date have primarily focused on multi‐joint free‐weight or semi‐guided exercises targeting the large lower‐body muscle groups involved in pedaling. Many studies have found beneficial effects on both muscle strength and cycling performance using the half squat as one of the primary exercises during RT in cyclists [24, 25, 26, 28, 29, 32, 35], whereas others have found beneficial effects performing full squats [16, 17, 37, 39]. It is worth noting that, in resistance‐trained non‐cyclist individuals, the full squat or parallel back squat have been reported to be more effective than partial range‐of‐motion alternatives (e.g., half squat) to increase muscle strength and even performance in functional tests (e.g., peak and mean power on the Wingate test, countermovement jump), inducing less pain and stiffness [52]. However, evidence to date has not compared the effects of these squat modalities specifically in cyclists. Moreover, complementary exercises can include the hip thrust, deadlift, and calf raise to comprehensively target the hip extensors, posterior chain, and plantar flexors [34, 37]. Notwithstanding, evidence comparing different exercise programs in cyclists is warranted to determine the optimal exercise selection.

Apart from the lower limbs, although scientific evidence is lacking in this regard, trunk musculature is expected to play a fundamental role in injury prevention, postural stability on the bike, and efficient force transmission from the lower limbs to the pedals. The configuration of core training differs substantially from the lower‐body RT described above, as the primary goal is to develop muscular endurance and neuromuscular control rather than maximal strength. Available evidence from cyclists and other endurance athletes suggests that core training should emphasize dynamic and isometric stabilization exercises (e.g., planks, side planks, abdominal crunches, dynamic and isometric side supports, bird‐dogs) performed with moderate‐to‐high repetition volumes or sustained holds, integrated into the training routine at a frequency of 2 to 3 sessions per week throughout the entire season [53]. However, evidence to date has failed to find beneficial effects of core training on cycling performance [42].

### 5.2 Training Intensity and Intent

Growing evidence suggests that RT for endurance athletes should diverge from traditional bodybuilding or general fitness approaches. Instead of seeking muscle hypertrophy indiscriminately as a primary goal, cyclists should aim to maximize strength gains while avoiding unnecessary non‐functional body mass gains; but allowing functional hypertrophy of the lower‐body musculature, which as noted above may accompany and even partly mediate the performance improvements observed with RT in cyclists [16, 17, 24, 25, 32]. In order to avoid the potential negative effects of excessive muscle hypertrophy (i.e., that resulting in reduced performance due to excessive increases in body mass), muscle function gains should be maximized through neural pathways (e.g., enhancing motor unit recruitment and increases in firing rates). To achieve these neural adaptations, both intensity and intent are paramount. Although hypertrophy can be induced across a wide spectrum of intensities, using relatively high loads (> 70% of 1RM) has been shown to produce superior strength gains [54]. Similarly, moderate‐to‐high loads in the range of 60%–85% of 1RM appear to maximize strength and neuromuscular adaptations in endurance athletes [55, 56], and indeed most intervention studies demonstrating performance benefits in cyclists have applied loads within this range [16, 17, 24, 27, 32]. It should be noted that some studies in non‐cyclists have reported that, when equated by relative volume load, similar strength gains can be obtained with low (40% 1RM) and moderate‐high loads (80% 1RM) [57]. However, a progression toward higher loads might be recommendable when possible. Conversely, current evidence in cyclists does not support an additional performance benefit of loads exceeding 85%–90% 1RM relative to the 60%–85% 1RM range. However, this should not be interpreted as evidence that such higher loads are contraindicated, since several effective interventions in cyclists have used loads of 85%–90% 1RM [12]. Moreover, every repetition should be performed with maximal concentric velocity intent, as this has been shown to enhance motor unit recruitment and rate of force development regardless of absolute load compared to controlled contractions [58, 59]. Thus, although these studies have not been specifically conducted in cyclists, the underlying neuromuscular mechanisms are likely applicable to this population.

### 5.3 Training Volume and Proximity to Failure

Apart from intensity, the volume of RT is also a critical factor. While high training volumes and training close to failure (with high relative effort) are key drivers of muscle hypertrophy, recent meta‐analytical data suggests that excessive training volume and proximity to failure may be counterproductive for athletes seeking functional strength [60]. Based on current evidence from RT literature in non‐cyclists, to maximize gains in muscle strength each session should include 3 to 5 multi‐joint lower‐body exercises, with 2 to 4 sets per exercise, accumulating approximately 6 to 20 sets per session [61]. Higher volumes are unlikely to provide additional performance benefits and may instead increase residual fatigue, potentially compromising subsequent endurance training quality. Between sets, a recovery period of 2 to 4 min is recommended to maximize strength gains when moderate levels of effort are maintained within each set [62].

Proximity to failure during each set also plays an important role in RT adaptations. This variable can be monitored through velocity loss, which serves as a real‐time indicator of neuromuscular fatigue: the greater the velocity loss during a set, the closer to muscle failure. Training to muscular failure substantially increases neuromuscular and metabolic fatigue, delays recovery, and maximizes hypertrophic responses [63]. It should be noted that several of the classical interventions that produced the clearest cycling performance benefits used traditional heavy RT performed with relatively high effort, often with sets of 4–12 repetitions and loads close to muscular failure [24, 25, 26, 27, 32]. Thus, training relatively close to failure has repeatedly proven compatible with meaningful cycling performance gains. Nonetheless, based on evidence derived from the general (non‐cyclist) RT literature, it can be argued that avoiding proximity to failure represents a reasonable strategy for managing residual fatigue and preserving the quality of subsequent endurance sessions. Thus, to manage residual fatigue and excessive non‐functional body‐mass gains, cyclists may consider limiting excessive velocity loss during each set. Indeed, research in non‐cyclists indicates that high velocity loss thresholds not only promote greater hypertrophy but may also impair improvements in explosive actions such as jumping, sprinting, and submaximal execution velocity [60]. Therefore, as demonstrated by Pareja‐Blanco and colleagues [64], terminating a set when a minimal velocity loss threshold is reached (e.g., < 20% or even < 10%) might be an effective strategy for maximizing gains in strength and power while avoiding non‐functional increases in muscle mass. Similarly, a recent study assessing the effect of concurrent training (i.e., running and RT) observed that a large velocity loss (40%) during RT induced the greatest gains in muscle mass compared to lower velocity loss thresholds. In turn, although all groups improved endurance performance indicators (e.g., maximal aerobic speed), gains diminished as velocity loss during RT increased, with the largest improvements observed with a threshold of ≤ 15% [65]. However, the effects of modifying proximity to failure or velocity loss during sets on cycling performance gains should be confirmed in studies conducted in cyclists.

### 5.4 Training Frequency

Regarding training frequency, current evidence suggests that a frequency of 2 RT sessions per week is an appropriate strategy for cyclists seeking to maximize strength adaptations while preserving compatibility with endurance training [12]. In trained individuals, strength gains can be achieved with relatively low training frequencies, whereas higher training frequencies do not appear to confer additional benefits when volume is equated [66]. Likewise, evidence of the minimum effective dose for strength development indicates that 2–3 weekly sessions are sufficient to induce significant improvements in 1RM, even with low training volumes, provided that the training stimulus (i.e., intensity) is adequately oriented toward maximal strength [67]. It should be noted that RT should also be ideally maintained during periods of high cycling load or competitive phases in which the primary objective is strength maintenance rather than further development [66, 67]. In this regard, a study in well‐trained cyclists showed that reducing RT frequency to 1 weekly session during the competition period was sufficient to preserve the gains in strength and muscle cross‐sectional area achieved during a preceding preparatory phase, while also enhancing relevant performance outcomes [25]. Thus, from a pragmatic point of view, 2 weekly sessions might be considered the preferred recommendation during phases of strength development in cyclists (e.g., during the preparatory period), whereas 1 weekly session seems to represent an effective and ecologically valid strategy for integrating RT during the competitive phase [25, 66, 67].

It is worth noting that to date most studies have examined the effects of adding RT to the usual cyclists' endurance training program [12]. However, evidence examining whether replacing, rather than adding, a cycling session with an RT session is beneficial in trained cyclists is scarce. One of the few studies to adopt this approach was that of Bastiaans et al. [19], who reported that replacing one third of the time of endurance training (~3 h per week) by explosive RT during 9 weeks prevented a decline in performance on a 30‐s cycling test without compromising gains in endurance performance. Notably, at the mid‐point assessment (4 weeks), the group that included RT maintained time‐trial and PPO performance while the endurance‐only group showed a decline suggesting an early protective effect of the concurrent approach [19]. Therefore, current evidence does not allow drawing firm conclusions as to whether replacing a cycling session with RT provides a superior, equivalent, or inferior overall stimulus compared with simply adding RT to the existing endurance program, or whether this depends on the cyclists' characteristics (e.g., training status, training volume).

### 5.5 Concurrent Training Considerations

Current literature on concurrent training suggests that separating endurance and RT sessions performed on the same day is likely advisable to reduce acute fatigue and potentially attenuate interference between training stimuli [68]. For instance, a meta‐analysis by Schumann et al. [69] observed lower gains in explosive strength with concurrent training compared with RT alone, but this interference effect disappeared when sessions were separated by at least 3 h regardless of session order.

Residual fatigue and substrate depletion induced by the first session may compromise the quality of the second session, which could, in turn, influence the adaptive response over time. In this context, if the primary goal is to enhance muscle strength/power, performing the RT session before cycling may be preferable; conversely, if the main objective is to maximize endurance performance or the quality of the cycling stimulus, the endurance session could be prioritized [70, 71]. Therefore, from a practical perspective, when both sessions must be completed on the same day, it seems reasonable to recommend a separation of ≥ 3 h between sessions when feasible, while acknowledging that this recommendation is derived largely from concurrent‐training research in non‐cyclist populations and should be interpreted cautiously until more sport‐specific evidence becomes available.

### 5.6 On‐Bike Strength (Torque) Training

The use of reduced cadence cycling as a means of increasing the muscular demands of each pedal stroke (known as torque training) initially emerged as a practical alternative to replace or complement traditional gym‐based RT. The rationale was that lowering cadence while maintaining power output would increase torque per revolution and thus stimulate strength‐oriented adaptations directly on the bike. A summary of studies applying on‐bike torque training interventions is shown in Table 2.

**TABLE 2:** Characteristics of main trials assessing the effects of on‐bike strength (torque) training in cyclists.

Authors	Total sample (groups)	Resistance training intervention			Main results
		Duration (frequency)	Exercises	Load	
Paton et al. (2009) [72]	18 [0F] (9 LLT; 9 HC)	4 weeks (2 sessions/week)	LLT: Single‐leg explosive jumps, low cadence cycling sprints 60–70 rpm. HC: Single‐leg explosive jumps, high cadence cycling sprints 110–120 rpm	LLT: Jumps: 20 reps/leg; maximal intensity Cycling: 5 × 30 s at 60–70 rpm (30 s rest); maximal intensity. HC: Jumps: 20 reps/leg; Cycling: 5 × 30 s at 110–120 rpm (30 s rest); maximal intensity	↑ PPO in both ↑ Mean PO at 4 mmol L^−1^ in both ↑ VO_2_max in both ↑ Cycling economy at 80% PPO in both ↑ Cycling economy at 50% PPO in LLT
Nimmerichter et al. (2012) [14]	17 [0F] (6 LLT; 6 HC; 5 C)	4 weeks (2 sessions/week)	LLT: Uphill intervals at RCP, low cadence 60 rpm. HC: Uphill intervals at RCP, high cadence 100 rpm	LLT: 6 intervals of 5 min; RCP power at 60 rpm. HC: 6 intervals of 5 min; RCP power at 100 rpm	↑ Uphill TT performance in LLT* ↑ Flat TT performance in both experimental groups*
Kristoffersen et al. (2014) [15]	22 [0F] (11 LLT; 11 FC)	12 weeks (2 sessions/week)	Pedaling cycle, low cadence intervals 40 rpm	LLT: 5 intervals of 6 min; 73%–82% HRmax at 40 rpm. FC: LLT: 5 intervals of 6 min; 73%–82% HRmax at freely chosen cadence	↑ VO_2_max in FC ↑ Mean PO at LT in FC ↑ VO_2_ in a 30‐min TT in FC ↑ Mean PO in a 30‐min TT in FC
Whitty et al. (2016) [73]	16 [0F] (8 LLT; 8 HC)	6 weeks (3 sessions/week)	LLT: Pedaling cycle, low cadence intervals 50 rpm. HC: Pedaling cycle, high cadence intervals 100 110 rpm	LLT: 4–6 intervals of 4 min; 70% MAP at 50 rpm. HC: 4–6 intervals of 4 min; 70% MAP at 100–110 rpm	↑ Freely chosen cadence on 60% Wmax in HC ↑ Wmax in both ↑ VO_2_max in both ↑ Gross efficiency in HC ↓ RPE in both **↑ TT distance in both (greater in LLT)** ↑ Mean PO in TT in both (greater in LLT)* ↑ Crank torque variables in both
Kristoffersen et al. (2019) [74]	28 [0F] (14 SST; 14 RT)	10 weeks (2 sessions/week)	SST: Seated cycling sprints 110–120 rpm. RT: Half‐squat, single‐leg leg press, hip flexion, toe raise	SST: 12 intervals of 6–8 s; maximal effort. RT: 3 sets of 4–8 reps; 4–8RM	↑ 6‐s PPO (fresh and post‐fatigue) in SST* ↑ **6‐s Mean PO** (**fresh** and post‐fatigue) **in SST**. RT only in post‐fatigue* ↑ 30‐s PPO in SST* ↑ 30‐s Mean PO in SST* ↑ Mean PO in a 5‐min TT in both ↓ 55‐m seated sprint time and ↑ Mean PO in SST* ↑ 1RM (greater in RT)* ↑ VO_2_max in RT ↑ Mean PO at 4 mmol L^−1^ in RT
Kordi et al. (2020) [75]	24 [7F] (13 IsoTORQ; 11RT)	6 weeks (2 sessions/week)	IsoTORQ: Cycling‐specific maximal isometric contractions at 45°, 90°, 135° knee angles on fixed ergometer, squat. RT: quat, deadlift, multi‐joint lifts and unilateral work (leg press, lunges, knee extension, leg curl, calf raises, core)	IsoTORQ: 1–4 sets of 3 reps per angle; maximal voluntary isometric torque +3 sets of 5 reps squat; 70%–75% 1RM. RT: 3–5 sets of 3–5 reps; 85%–95% 1RM	↑ PPO in IsoTORQ* ↑ Cycling‐specific isometric strength in IsoTORQ* ↑ Maximal torque in IsoTORQ
Valenzuela et al. (2021) [49]	18 [0F] (8 SST; 10 VBRT)	7 weeks (2 sessions/week)	SST: Cycling sprints (standing). VBRT: Squat, lunge, hip thrust	SST: 3 sets × 4 intervals × 6 s; all‐out, highest gear (53 × 11) VBRT: 3 sets of maximum repetition to 10% VLoss; Optimal load (~65% 1RM)	↓ Fat mass in VBRT ↑ BMC in VBRT ↑ 1RM in VBRT ↑ VT in SST ↑ RCP in VBRT ↑ Mean PO in an 8‐min TT in both
Hebisz et al. (2024) [76]	24 [24F] (12 LLT; 12 C)	8 weeks (2 sessions/week)	1 day cycling sprints 50–70 rpm (SIT), 1 day cycling intervals 50–70 rpm (HIIT)	SIT: 8–12 intervals of 30 s; maximal effort at 50–70 rpm. HIIT: 4–6 intervals of 4 min; maximal effort at 50–70 rpm	↑P_max_ in LLT* **↑VO**_**2**_ max in both (greater in LLT)* ↑VT1 in LLT ↑VT2 in LLT ↑VEmax in LLT*
Barranco‐Gil et al. (2025)^a^ [16]	25 [0F] (12 HLT; 13 C)	10 weeks (2 sessions/week)	Maximal pedaling cycles on 6% slope (40–50 rpm)	5 intervals of 7 pedal cycles; heavy gear adjusted so force = 70% MDF (40–50 rpm)	↑ 1RM in HLT* ↑ Pedaling‐specific strength in HLT* ↑ Mean PO and PPO in the Wingate test in HLT* ↑ CSA in HLT*
Pallares et al. (2025)^a^ [17]	25 [0F] (12 HLT; 13 C)	10 weeks (2 sessions/week)	Maximal pedaling cycles on 6% slope (40–50 rpm)	5 intervals of 7 pedal cycles; heavy gear adjusted so force = 70% MDF (40–50 rpm)	↑ 1RM in HLT* ↑ Pedaling‐specific strength in HLT* ↑ VT in HLT ↑ MAP in HLT ↑ CSA in HLT
Vicari et al. (2026) [77]	19 [3F] (11 RT; 8 HLT)	12 weeks (2 sessions/week)	RT: Half squat, deadlift, dumbbell lunges, Bulgarian squat. HLT: Cycling sprints at low cadences on 4%–5% slope	RT: 2–4 sets of 4–12 reps; 65%–85% 1RM. HLT: 3–4 sets of 6–8 intervals of 1 s; maximal efforts	↑ PPO in the Wingate test in RT* ↑ Mean PO in the Wingate test in RT ↑ ATP in RT*
de Pablos et al. (2026) [78]	46 [0F] (16 HLT; 15 LLT; 15 C)	10 weeks (2 sessions/week)	HLT: Maximal pedaling cycles on 6% slope (40–50 rpm). LLT: Pedaling cycle, low cadence intervals 50 rpm	HLT: 5 intervals of 7 pedal cycles; heavy gear adjusted so force = 70% MDF (40–50 rpm). LLT: 5 intervals of 4 min; 70% MAP = 35% MDF at 50 rpm	↑ Mean PO and PPO in the Wingate test in HLT* ↑ MAP in HLT ↑ VT1 in HLT ↑ RCP in HLT ↑ CSA in HLT*

*Note:* Results in the Main results column are those that showed significant within‐group changes, and those in bold font and marked with an asterisk also showed significant between‐group differences (*p* < 0.05).

Abbreviations: 1RM, one repetition maximum; ATP, anaerobic threshold power; BM, body mass; BMC, bone mineral content; BMI, body mass index; C, control; CSA, cross‐sectional area; FCC, freely chosen cadence; G, group; HC, high cadence; HIIT, high intensity interval training; HLT, high load torque; IsoTORQ, isometric torque training; LLT, low load torque; MAP, maximal aerobic power; MDF, maximal dynamic force; min, minutes; NR, not reported; OPL, optimal power load; P_max_, maximum power; PO, power output; PPO, peak power output; RCP, respiratory compensation point; rpm, revolutions per minute; RT, resistance training; RTD, rate of torque development; SIT, sprint interval training; SST, short‐sprint training; TS, total sessions; VE, maximal pulmonary ventilation; VT, ventilatory threshold; Wmax, peak power.

^a^ These studies pertain to the same trial.

The initial torque training methodologies integrated low cadence into prolonged aerobic sessions or moderate‐intensity interval work (e.g., 4–6 min intervals performed at 40–60 rpm and submaximal intensities) [14, 15]. Evidence on their effectiveness is mixed. For instance, low‐cadence interval training at moderate intensity improved uphill time‐trial performance significantly more than high‐cadence training in one study [14], and both low‐ and high‐cadence groups improved time‐trial performance in another, with the low‐cadence group showing greater gains [73]. However, studies in veteran cyclists reported negligible effects on endurance performance, with adaptations largely restricted to shifts in preferred cadence rather than increases in maximal force production [15]. Critically, where performance improvements have been observed with submaximal low‐cadence protocols, these are likely attributable to other metabolic adaptations rather than neuromuscular strength development. Recent evidence demonstrates these interventions impose relatively low mechanical demands, generally remaining well below the force levels associated with effective strength loading in RT [54, 79]. In contrast, sprint‐based low‐cadence interventions, which impose higher force demands per pedal stroke, showed more consistent improvements in PPO, cycling economy, and power at lactate threshold [72], although the relative mechanical load was not quantified at the time.

However, recent work quantifying pedaling force relative to maximal dynamic force (MDF) demonstrates that these “classic” submaximal torque training interventions rarely exceed 40%–50% MDF, even under deliberately constrained cadence conditions [79]. This relative intensity is clearly below the range generally associated with effective strength loading in RT [54], reinforcing the conclusion that traditional low‐load torque approaches do not provide sufficient mechanical overload for strength development. Such findings help explain the modest and inconsistent outcomes reported in those torque‐oriented interventions.

Building on this insight, some methodological variations have attempted to increase the effectiveness of on‐bike strength‐oriented stimuli by introducing higher‐intensity efforts. For instance, sprint‐based interventions performed at low cadence have been shown to improve PPO and selected performance variables in trained cyclists [74]. Similarly, performing on‐bike sprints has been associated with improvements in PPO, ventilatory thresholds, and time‐trial performance in professional cyclists [49]. However, in these studies, pedaling intensity was not prescribed relative to maximal force capacity, and therefore the actual mechanical stimulus remained unknown. In parallel, other approaches have explored whether high‐intensity force‐oriented stimuli applied directly on the bike can induce meaningful neuromuscular adaptations. For example, cycling‐specific isometric RT performed at fixed joint positions has been shown to increase PPO and neuromuscular function in trained cyclists [75]. Although distinct from torque‐based approaches, these findings reinforce the notion that the bicycle can serve as an effective medium for strength development when sufficiently high mechanical loads are applied.

A methodological shift occurred when our group validated a method to assess MDF during cycling, which enables pedaling intensity to be expressed relative to MDF in a manner conceptually analogous to prescribing RT based on %1RM [80]. This represented a decisive progression, as it enabled objective and individualized control of mechanical load during on‐bike strength‐oriented sessions. Within this framework, high‐load torque interventions have been implemented using short maximal efforts (e.g., 5 sets of 7 all‐out pedaling cycles performed at ~70% MDF with 4‐min recovery periods) that correspond to an intensity of approximately 70% MDF [16, 17]. This approach induced substantial improvements in pedaling torque production, sprint performance, and quadriceps cross‐sectional area [17], as well as in selected endurance indicators such as maximal aerobic power and ventilatory thresholds, with the magnitude of improvements comparable to those observed with conventional off‐bike squat training performed at a matched relative load [16]. The role of relative load during torque training has been further clarified in a recent three‐arm trial comparing high‐load (70% MDF) and low‐load torque (35% MDF) with a control condition [78]. Although no significant differences were found between low‐ and high‐load conditions (despite moderate effect sizes favoring the high‐load condition for some outcomes such as the Wingate performance), only the high‐load condition induced consistent within‐group improvements in Wingate performance, maximal aerobic power, ventilatory thresholds and quadriceps cross‐sectional area, whereas the low‐load protocol, representative of traditional torque practices, showed limited or inconsistent effects.

Taken together, evidence overall suggests that on‐bike high‐load torque training can provide beneficial effects on pedaling torque production capacity and cycling performance parameters, at least when relative intensity is high (e.g., ≥ 70% MDF). Notably, early evidence suggests that these benefits may be similar to those induced by conventional off‐bike (gym‐based) RT if mechanical variables are matched, which opens the possibility of integrating strength‐oriented stimuli directly on the bike when conventional RT is not feasible. Whether whole‐body gym‐based training confers additional benefits beyond those achievable through on‐bike methods (e.g., through upper‐body conditioning and attenuating muscle and postural imbalances) remains an open question warranting further investigation. Several practical limitations should also be considered before high‐load on‐bike torque training is widely adopted. First, quantifying MDF currently requires specific equipment and testing protocols that limit its feasibility outside specialized laboratory or sport science settings. Second, performing maximal or near‐maximal efforts at very low cadence may raise safety considerations related to joint and tendon loading. However, no major safety signal has been reported in the few existing trials to date, with cyclists reporting similar levels of pain and stiffness with on‐bike high‐load torque training compared to conventional RT [17]. Third, the available trials have been conducted predominantly in young, well‐trained male cyclists, so generalizability to other populations (e.g., recreational, female or master cyclists) remains unknown. Finally, it remains unknown whether on‐bike torque training confers the same bone‐health benefits as gym‐based RT, since it lacks the axial and multi‐joint loading patterns (i.e., through the trunk and upper‐body during free‐weight squatting or hip‐thrust exercises) that may underlie some of the benefits on bone mineral density described above.

## 6 Perspectives

While cyclists often avoid RT due to concerns over residual fatigue and hypertrophy, strong evidence supports its benefits on different performance indicators (e.g., efficiency/economy, time to exhaustion, durability) and actual cycling performance (time trial and sprint performance). A summary of the benefits of strength training for cyclists and practical recommendations for its implementation can be found in Figure 1. Although muscle hypertrophy may occur, body mass typically remains stable due to concomitant fat mass reduction; furthermore, weight gain seems to be functionally offset by substantial improvements in relative and particularly absolute power output. Recent but still limited evidence further indicates that high‐load on‐bike torque training may also elicit relevant benefits on some cycling performance indicators, offering a potentially viable strategy when access to RT facilities is limited. Moreover, it is worth noting that beyond performance, RT might improve postural control and mitigate the low bone mineral density prevalent in competitive cyclists, although further research is warranted in this regard. However, adaptations to RT are highly dependent on precise programming, where intensity, proximity to failure, and recovery periods are critical variables. While this review provides evidence‐based guidelines for its practical implementation, some of the recommendations are based on non‐cyclist populations, and therefore further research involving elite‐level cyclists is necessary to refine these recommendations.

**FIGURE 1:**
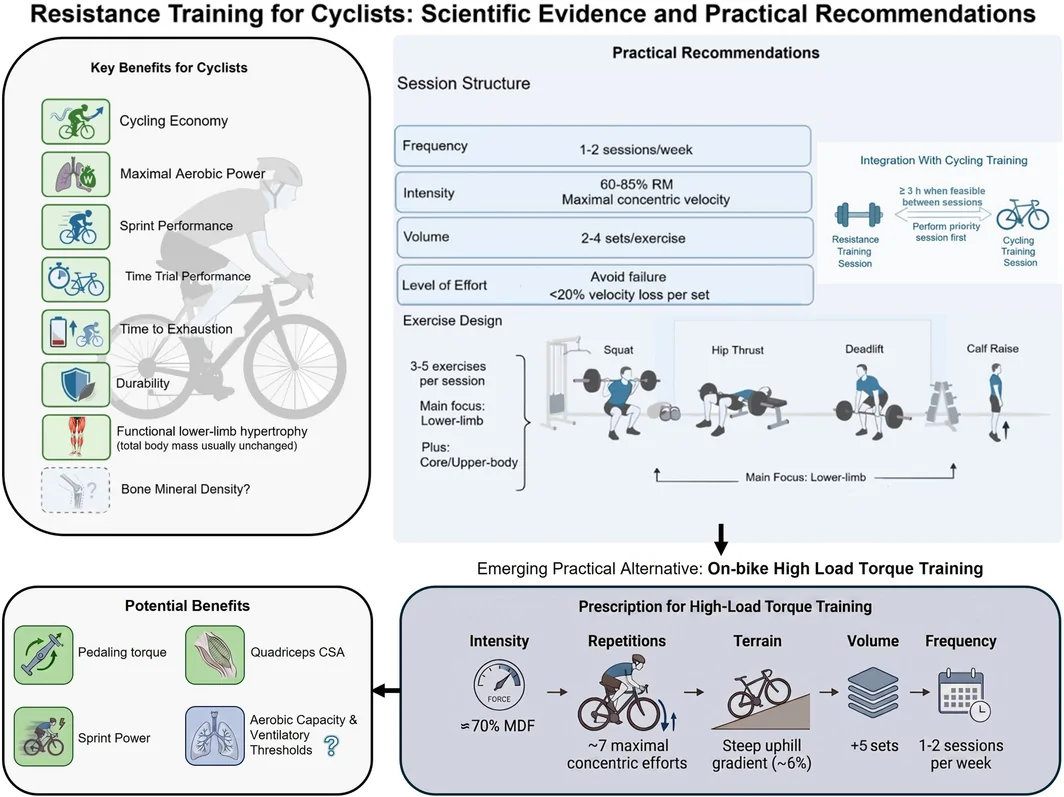
Summary of the effects of resistance training and high‐load torque training in cyclists, and evidence‐based practical recommendations for their implementation. Of note, some of these are pragmatic recommendations derived by both cyclist‐specific and broader evidence in the general population, and not all parameters have been directly established as optimal for cyclists.

## Funding

P.L.V. is supported by a postdoctoral contract granted by Agencia Estatal de Investigación (RYC2024‐048275‐I).

## Conflicts of Interest

The authors declare no conflicts of interest.

## Data Availability

Data sharing not applicable to this article as no datasets were generated or analysed during the current study.
